# Exome sequencing reveals a de novo PRKG1 mutation in a sporadic patient with aortic dissection

**DOI:** 10.1186/s12881-018-0735-1

**Published:** 2018-12-22

**Authors:** Wenwen Zhang, Qian Han, Zhao Liu, Wei Zhou, Qing Cao, Weimin Zhou

**Affiliations:** 1 Department of Vascular Surgery, The Second Affiliated Hospital of Nanchang University, No 1#, Minde Road, Nanchang, China; 2 Key Laboratory of Molecular Medicine of Jiangxi Province, Nanchang, Jiangxi China; 3 Department of Vascular Surgery, Nanjing Drum Tower Hospital, the Affiliated Hospital of Nanjing University Medical School, Nanjing, Jiangsu China

**Keywords:** Aortic dissection, *PRKG1* gene, Whole-exome sequencing, Binding motif

## Abstract

**Background:**

Thoracic aortic aneurysm and dissection (TAAD) is a common condition associated with high mortality. It is predominantly inherited in an autosomal dominant manner with reduced penetrance and variable expression. The genetic basis of the majority of TAAD cases remains unknown.

**Case presentation:**

We described a 53 years old male presented with abdominal aortic dissection as well as aortic tortuosity. To investigate the genetic basis of the clinical presentation, whole-exome sequencing was performed. Exome sequencing identified a de novo heterozygous undescribed mutation in the *PRKG1* gene (NM_001098512.2: c.1108 G > A), predicted to cause the missense change p.Gly370Ser in the ATP binding motif of the protein. This mutation was not reported in the dbSNP, 1000 Genome Project, and Exome sequencing databases. Furthermore, the Glycine370 residue of PRKG1 is highly conserved among various species and it is predicted to be damaging by multiple in silico programs, suggesting that this substitution may cause a major disruption of protein function. To our knowledge, this is the second reported mutation locus of *PRKG1* accounting for the disease.

**Conclusions:**

Our study expands the mutation spectrum of *PRKG1* and clinical phenotype of mutation-carriers. Screening for *PRKG1* mutations should be considered in patients with unexplained aortic disease, and identification of the causative gene will aid in individualized, gene-tailored management.

## Background

Thoracic aortic aneurysm and dissection (TAAD) remains a significant clinical challenge due to its high morbidity and mortality [1]. Previous studies have demonstrated that approximately 20% of TAAD patients have a family history, indicating a genetic component to the disease [2]. It is predominantly inherited in an autosomal dominant fashion with reduced penetrance and variable expression, showing significant genetic (> 20 genes) and clinical (location, severity and age of onset) heterogeneity [3]. TAAD patients caused by different genes have distinct clinical courses and risk of rupture or dissection, influencing intervening measure and timing [4]. The identification of specific mutated genes in patients with TAAD is therefore crucial because it permits individualized, gene-tailored therapy.

The majority of genes associated with the development of TAAD encode proteins involved in the extracellular matrix maintenance, smooth muscle cell (SMC) contraction or metabolism, or canonical transforming growth factor-β signaling pathway [5]. Dysfunction of SMC contractile apparatus impairs resilience of aorta in the context of pulsatile blood flow and shear stress. Accordingly, mutations in genes encoding smooth muscle-specific isoform of α-actin (*ACTA2*) and myosin heavy chain dimer (*MYH11*) are responsible for roughly a quarter of familial TAAD patients [6, 7]. Mutations in *MYLK* are another rare cause of familial TAAD [8]. *MYLK* encodes smooth muscle myosin light chain kinase, which drives SMC contraction through phosphorylation of the regulatory light chain on the thick filaments.

In 2013, a single heterozygous mutation in the gene encoding cGMP-dependent protein kinase 1 (*PRKG1*; protein PKG-1; chromosomal 10q11.2-q21.1) was demonstrated to cause TAAD [9]. PKG-1 is activated on binding of cGMP and plays an important role in SMC relaxation. The causal mutation, pArg177Gln, is located in the high-affinity cGMP binding site, and such alteration in this domain abolished binding of cGMP. Paradoxically, the mutation resulted in the enzyme being constitutively active even in the absence of cGMP, suggesting the mechanism of gain-of-function. Individuals harboring this mutation have aortic dissections at a relatively young age (15–51 years) at diameters ranging 4.3 to 5.7 cm. Aortic tortuosity and hypertension are also noted in some mutation-carriers.

Here, we identified an individual with abdominal aortic dissection characterized by tortuosity, as well as congenital visual impairment caused by keratoconus. Whole-exome sequencing revealed a heterozygous sequence variation in *PRKG1* (c.1108 G > A, p.Gly370Ser). The mutation lies in a conserved motif with pivotal function, and multiple lines of evidence support its pathogenic role. To our knowledge, this is the second reported mutation locus of *PRKG1* accounting for the disease.

## Case presentation

**Fig. 1:**
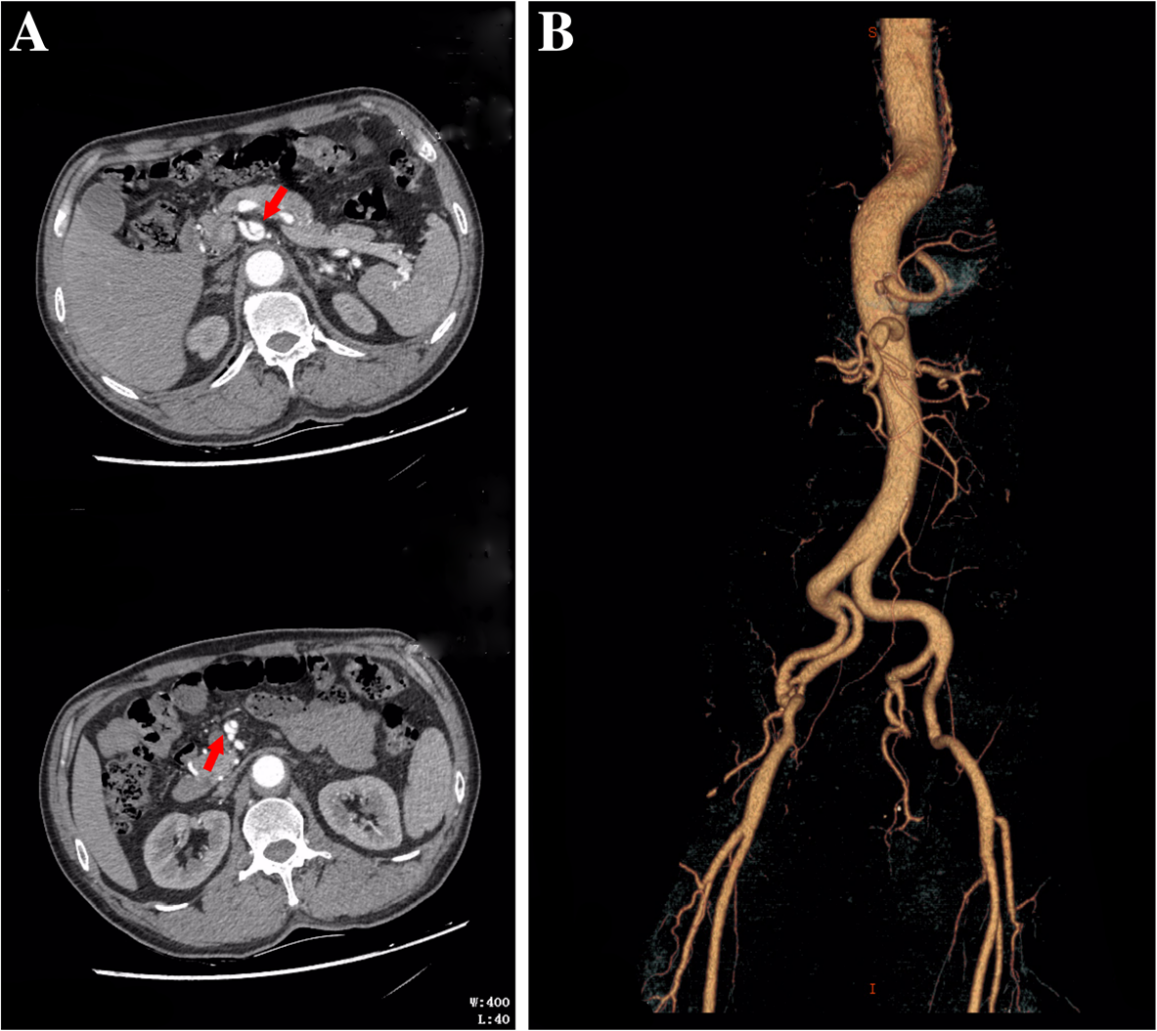
Radiographic findings of the patient. **a** Multi-slice computed tomography shows abdominal aortic dissection with the celiac artery and the superior mesenteric artery (Red arrow) involved. **b** 3D-reconstructed computed tomography angiogram shows tortuosity of abdominal and iliac aorta

The index patient was a male of 53 years who was referred to our department complaining of abdominal pain, which failed to subside after administration of antispasmodic agents. Computed Tomography Angiography (CTA) showed abdominal aortic dissection originating distal to the renal artery and extending to the iliac arteries, with the celiac artery and the superior mesenteric artery involved (Fig. 1a). Tortuosity of aorta was also observed (Tortuosity was defined as the presence of at least two bends > 70° proximal to the target lesion or one proximal bend > 90°) (Fig. 1b). His medical history was significant for hypertension for 4 years treated with Ca antagonists. He had ischemic heart disease, including history of acute myocardial infarction at age 51 with no stents implanted. His past medical history also included moderate scoliosis diagnosed at age 10.

On physical examination, clinical features reminiscent of connective tissue disorders were not remarkable, except for congenital visual impairment. Subsequent corneal topography measurements were taken and keratoconus was diagnosed. Family history of aorta diseases was negative, as his parents displayed normal aorta on contrast-enhanced CT. The family provided written informed consent for clinical and molecular analyses, and the research protocol was approved by the Institute Review Board of the Second Affiliated Hospital of Nanchang University.

The patient was first diagnosed as arterial tortuosity syndrome (ATS), due to strikingly overlapped clinical features (aorta dissection, tortuosity and keratoconus) [10]. The coding exons and intronic boundaries of the *SLC2A10* gene was first screened by direct Sanger sequencing, with no causative mutation found in the gene.

To investigate the genetic basis of the disease, whole-exome sequencing was performed as previously described [11]. Genomic DNA was captured using the Agilent SureSelect V6 enrichment kit and sequenced on an Illumina HiSeq 2500 as 150-bp paired-end runs. In accordance with the Genome Analysis ToolKit’s (GATK) best practices guidelines, we mapped sequence reads to the human reference genome (hg19) using the Burrows-Wheeler Aligner (v.0.7.8), removed duplicate reads (Picard v.1.111), and identified SNPs and Indels (SAMtools v.1.0). ANNOVAR (v. 2015Dec14) was utilized to annotate the detected variations.

On average, 99.8% of short reads were mapped, reaching an average ~ 130-fold depth of coverage. 99.6% of targeted regions were covered by at least 10 unique reads. A summary of the filtering strategy of this study is presented in Table 1. Exome data yielded 47 likely pathogenic variants after sequential filtering process. Among them, we focused on variants of known causal genes or genes implicated in aorta diseases. Finally, an unreported heterozygous c.1108 G > A (p.Gly370Ser) missense mutation in the 10th coding exon of the *PRKG1* gene (NM_001098512.2) was identified.

**Table 1:** Filtering strategy of the patient

Filtering criteria	Number of variants
Total number of variants	169,956
Missense, nonsense, splice-site or frameshifit variants	10,363
MAF < 1% in the 1000G, ESP, ExAC	520
Absence in our in-house databases	245
Prediction in silico (SIFT, Polyphen2, MutationTaste)	61
CADD score > 15	47

**Fig. 2:**
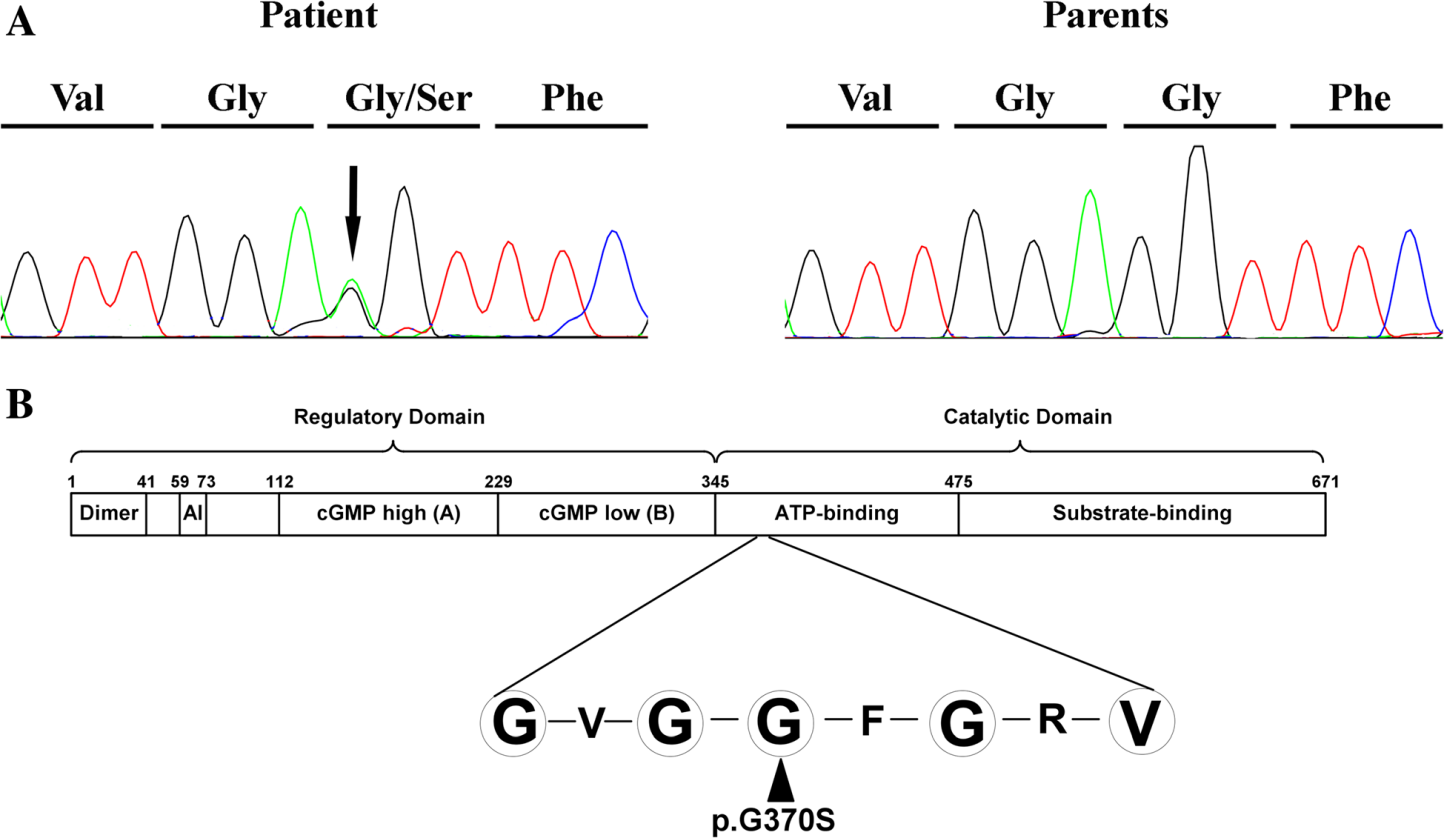
Sanger sequencing and mutation location. **a** The DNA sequencing chromatograms represent the patient and his parents. The corresponding encoded amino acid is shown above the chromatograms and the arrow denotes the mutated nucleotide. **b** A graphic illustration of the functional domain structure of PKG-1α and the glycine-rich GXGGXXGXV motif where the mutation p.Gly370Ser is located, as indicated by triangle. At the N-terminus, the dimerization domain is depicted. AI = the auto-inhibition domain; cGMP high (called A) = high-affinity binding domain for cGMP; cGMP low (called B) = low-affinity binding domain for cGMP

Bidirectional Sanger sequencing confirmed the mutation in the patient. By employing short tandem repeat multiplex assay, we confirmed the biological association of the father and mother with the patient. Its absence in both parents validated the de novo status of the mutation (Fig. 2a). The c.1108 G > A variant was absent from dbSNP144, the 1000G, ESP, ExAC and our in-house variant databases. Furthermore, this variant was predicted as damaging according to online prediction tools such as SIFT, Polyphen2 and Mutation Taster. Moreover, the CADD C-score of the mutation was 33, strongly predicting that the p.Gly370Ser as deleterious (scores > 15 are deleterious).

The main gene product of *PRKG1* expressed in the aorta, PKG-1α, is a homodimer with the N-terminal regulatory and C-terminal catalytic domains (Fig. 2b) [12]. Located in the ATP binding region, the glycine-rich GXGGXXGXV motif is capable to bind the adenine ring of ATP by forming a hydrophobic pocket [13]. The variant identified in this study is located in the third highly conserved glycine residue. In addition, the replacement of hydrophobic glycine residue by hydrophilic serine may cause the conformational change of the pocket, disrupting its binding capability and thereby impairing protein function. According to the latest American College of Medical Genetics and Genomics (ACMG) guidelines for the interpretation of sequence results, this variant in *PRKG1* gene fulfills PM1, PM2, PP3 and PS2 and is therefore regarded as likely pathogenic [14].

## Discussion and conclusions

TAAD is a heterogenous monogenic disorder characterized by overlapping manifestations and variable expression. It is therefore challenging to identify the underlying gene defect just based on its clinical features. Conventional Sanger sequencing is time-consuming and laborious if multiple disease-causing genes exist. In this study, by exome sequencing we successfully identified the culprit mutation of in a patient who first had a diagnosis of ATS, given the particularly fitting clinical features. Molecular analysis revealed a heterozygous de novo missense mutation c.1108 G > A (p.Gly370Ser) in the *PRKG1* gene.

Multiple lines of evidence support this variant as a pathogenic variant causing the patient’s clinical presentation. First, no missense variants have been reported in the public variation databases at this position. Second, the p.Gly370Ser is located within the glycine-rich ATP binding region, a highly-conserved motif across eukaryotic protein kinases. The motif functions as a hydrophobic pocket capable of binding the adenine ring of ATP, and substitution of glycine for hydrophilic serine at this position is unlikely to be tolerated. Third, in silico analysis using multiple online tools shows consistently that the p.Gly370Ser substitution is predicted to be damaging to protein function. Amino acid property probably plays a key role in the correct folding of this region into the hydrophobic pocket. It is notable that not only is residue 370 the hydrophobic glycine, but that the surrounding residues are also highly conserved non-polar residues [13]. The substitution of Gly370 by the polar serine is likely to cause local structure instability. Taken together, it is very likely that the p.Gly370Ser substitution will cause disruption of the hydrophobic pocket, and, since this has been shown to be indispensable for interaction with ATP, perturbation of protein function.

Guo et al. identified a recurrent mutation, p.Arg177Gln, in four unrelated family of TAAD [9]. The mutation segregated with the disease in each family and was not present in controls. The p.Arg177Gln is located in the high-affinity cGMP binding site, another conserved domain with pivotal function. In vitro function studies demonstrated that it resulted in constitutive kinase activity even in the absence of cGMP, indicating gain-of-function for the mutation. Mutation-carriers presented with either thoracic aortic dissection or aortic root dilatation, with thoracic aortic disease fully penetrate in those aged over 18 years old. Some patients also suffered from abdominal aortic dissection and showed aorta tortuosity, as reported in our case. Gago-Diaz et al. investigated the molecular defect in a large TAAD pedigree and found the same mutation, suggesting that the p.Arg177Gln may be a mutation hotspot [15]. The penetrance of TAAD in this family was nearly complete, except for a 33-year-old female. Apart from aortic features, other extra-aortic connective tissue manifestations were also found in a subset of patients, which includes scoliosis, wrist and thumb signs, skin striae, myopia, pectus carinatum/excavatum deformity, chest asymmetry. But none of them fulfilled diagnostic criteria of the main connective tissue syndromes.

Ocular problem constitutes an important feature in a considerable proportion of patients harboring mutations in TAAD disease-causing genes [16]. For example, ectopia lentis and myopia were frequently observed in *FBN1* mutation-carriers; myopia in *SKI*; mydriasis in *ACTA2* [17]. The revised Ghent nosology puts more weight on ectopia lentis in the diagnosis of Marfan syndrome caused by *FBN1* mutation [18]. The occurrence of ocular problem in TAAD patients can to some degree aid in the recognition of the underlying genes. A special form of ocular disorders, keratoconus, is very rare in TAAD patients. ATS is an uncommon autosomal recessive disorder characterized by widespread arterial involvement with tortuosity, elongation, and aneurysms of the large and middle-sized arteries, with some patients exhibiting keratoconus phenotype [10]. The inheritance model and clinical features of our case made us suspect the diagnosis of ATS, while genetic testing of *SLC2A10* yielded negative results. Our study is the first to show the phenotype of keratoconus in *PRKG1* mutation-carriers. However, whether it is associated with *PRKG1* mutation or just accidental finding requires further research.

Based on the anatomic location, aortopathy is subdivide into abdominal and thoracic aortic lesions. Abdominal aortic lesions are more common and tend to occur at later age. They seem to be multifactorial and associated with common cardiovascular risk factors. So far, no disease-causing variants have been identified to account for abdominal aortic diseases. However, thoracic aortic lesions have a greater heritability and often show family clustering. Indeed, a substantial proportion of them are manifestations of part of Mendelian connective tissue disorder. Apart from thoracic involvement, some patients also exhibit non-thoracic lesions, such as abdominal and intracranial diseases. Their features include early-onset and a lack of common cardiovascular risk factors. Their underlying genetic architecture often warrants further investigation.

In conclusion, we report a novel case affected with aortic dissection and have identified a novel likely pathogenic variant in the ATP binding domain of *PRKG1*, which provides further evidence for the role of this gene in causing TAAD. Adding to previous reports, our study underlines the need to include this gene among the candidate genes tested in patients affected with unexplained aortic disease. Identification of the causative gene allows identification of additional risky family members and gene-based management of the carriers.

## Abbreviations

ACMG: American college of medical genetics and genomics
ATS: Arterial tortuosity syndrome
CTA: Computed tomography angiography
GATK: Genome analysis toolkit
SMC: Smooth muscle cell
TAAD: Thoracic aortic aneurysm and dissection
